# Chyme Reinfusion Reducing the Postoperative Complications After Definitive Surgery for Small Intestinal Enteroatmospheric Fistula: A Cohort Study

**DOI:** 10.3389/fnut.2022.708534

**Published:** 2022-02-21

**Authors:** Weiliang Tian, Risheng Zhao, Xin Xu, Yunzhao Zhao, Shikun Luo, Shen Tao, Zheng Yao

**Affiliations:** ^1^Department of General Surgery, Jinling Hospital, Nanjing, China; ^2^Department of General Surgery, Jiangning Hospital, Nanjing, China; ^3^Department of General Surgery, Nanjing 83 Hospital, Nanjing, China

**Keywords:** chyme reinfusion, enteroatmospheric fistula, outcomes, surgery, recurrent fistula

## Abstract

**Purpose:**

This study is aimed to reveal the role of preoperative chyme reinfusion (CR) in reducing the complications occurring after definitive surgery (DS) for small intestinal enteroatmospheric fistula (EAF).

**Methods:**

In this study, from January 2012 to December 2019, the patients with small intestinal EAF and receiving a definitive surgery were recruited. Depending on whether the CR has been performed, these patients were divided into either the CR group or the non-CR group. Then, propensity scores matching (PSM) was used to further divide these patients into the PSM CR group or the PSM none-CR group. The clinical characteristics exhibited by the groups were analyzed, and the effect of preoperative CR was investigated.

**Result:**

A total of 159 patients were finally recruited with 72 patients in the CR group and 87 patients in the non-CR group. The postoperative complications were manifested in a total of 126 cases (79.3%). There were 49 cases in the CR group, and 77 cases in the non-CR group. CR was associated with the occurrence of postoperative complications (multivariate odds ratio [*OR*] = 0.289; 95% *CI*: 0.123–0.733; *p* = 0.006). After 1:1 PSM, there were 92 patients included. The postoperative complications were observed in 67 out of these 92 patients. There were 26 patients in the PSM CR group, and 41 patients in the PSM non-CR group. CR was associated with postoperative complications (multivariate *OR* = 0.161; 95% *CI*: 0.040–0.591; *p* = 0.002). In addition, CR played a role in reducing the recurrence of fistula both before (multivariate *OR* = 0.382; 95% *CI*: 0.174–0.839; *p* = 0.017) and after (multivariate *OR* = 0.223; 95% *CI*: 0.064–0.983; *p* = 0.034) PSM. In addition, there is a protective factor at play for those patients with postoperative ileus before (multivariate *OR* = 0.209; 95% *CI*: 0.095–0.437; *p* < 0.001) and after (multivariate *OR* = 0.222; 95% *CI*: 0.089–0.524; *p* < 0.001) PSM. However, the relationship between CR and incision-related complications was not observed in this study.

**Conclusion:**

Preoperative CR is effective in reducing postoperative complications after definitive surgery was performed for EAF.

## Introduction

Falling into the category of enterocutaneous fistula (ECF), enteroatmospheric fistula (EAF) is defined as the passage between the gastrointestinal (GI) tract and the atmosphere (1). Given a large amount of intestinal juice leaked from the fistula, the flow into the distal small intestine is obstructed, which leads to a series of functional and structural changes occurring to the bypassed intestine, such as villous atrophy, smooth muscle weakness, and endocrine disturbances (2, 3). In this circumstance, it is often necessary to perform definitive surgery (DS) for EAF after the formation of a frozen abdomen (4). However, this is a highly complex process with high incidence of complications (5), which might be closely related to the intestinal function (5).

Chyme reinfusion (CR) refers to an enteral nutrition technique that can be applied to artificially re-establish the small bowel continuity through an extracorporeal circuit of chyme, which activates the definitive GI function (6). This process proves effective not only in improving the nutritional status, organ functions, and the digestive tract function of distal stoma or fistula (7, 8), but also in maintaining the pathological changes caused by the disuse of the digestive tract (9, 10). In theory, the post-CR restoration of intestinal function might play a role in reducing the occurrence of postoperative complications. However, this appears not to reach a significant extent. Therefore, it is hypothesized in this retrospective study that CR is associated with the improved outcomes of postoperative DS.

## Materials and Methods

This is a retrospective cohort study performed at two tertiary hospitals, where there are hundreds of patients with refractory intestinal fistula transferred annually. This study was granted approval from the institutional review boards at these two hospitals. All methods were adopted in strict accordance with the applicable guidelines and regulations. Informed consent was obtained from all of the participants in this study.

### EAF Treatment and CR

The treatment of EAF in our study consists of three stages. During the first stage, the infection was subjected to control, with total enteral nutrition and CR implemented steadily to result in the expected ventral hernia *via* an epidermal skin graft. CR was performed for each patient with source control in place. Chyme was sucked out into the collection bottle using a negative pressure drainage tube. Then, it was reinfused without being filtered by an enteral nutrition pump into the downstream digestive tract *via* a 16F Foley tube as the reinfusion tube, which was fixed by the non-woven medical tape. In the daytime, this process was carried out once every 1–2 h; at night, it was carried out every 2–4 h. The speed of reinfusion was calculated, so as to ensure that the digestive secretions collected during each hour could be taken as a sufficient input during the next hour.

The second-stage treatment was performed after the above-mentioned goal was achieved. During this stage, the patients were transferred to their local community health service centers or local hospitals capable of continuing the treatment for recuperation and physical recovery, along with at least 4 months of recuperate.

A DS for EAF was performed during the third stage. With the criteria for DS satisfied, the patients were transferred to our institution again for DS. Beyond this stage, the patients would make full recovery and get discharged after the successful treatment.

Throughout the course of treatment, CR was recommended until DS. CR was carried out steadily when each patient was transferred to the local institute. However, in clinical practice, there remained a large number of patients failing to continue with CR in the second stage.

### Population

The patients with small intestinal EAF who had received DS from January 2012 to December 2019 were recruited in this study. The exclusion criteria are as follows. First, the patients are aged under 18 years of age. Second, the patients with enteral nutrition providing less than 60% of the nutritional needs. Third, the patients who develop inflammatory bowel disease (IBD). Fourth, the patients manifest upper GI fistula, colon fistula, pancreatic fistula, or pancreatitis, which make it difficult to perform the surgery. Last, patients with incomplete data.

Those patients with a cumulative duration of over 1 month for CR during the second stage were assigned into the CR group, while the remaining patients were assigned into the Non-CR group. Then, the characteristics of the patients in the two groups were reviewed and analyzed.

### Primary Outcome and Secondary Outcomes

The patients were followed-up until discharged from hospital. The primary outcome was various postoperative complications. Each major surgical complication was considered separately as a secondary outcome.

### DS for EAF

These patients were still followed-up during the treatment performed at the local institution. When the criteria set for DS were satisfied, the patients were transferred to our institution, with a DS scheduled. The DS criteria are detailed as follows. First, C-reactive protein (CRP), white blood cell (WBC), and procalcitonin (PCT) are kept normal for more than 1 month. Second, body mass index (BMI) ≥ 18.0 kg/m^2^ and normal physical strength is maintained. Third, hemoglobin ≥ 110 g/L. Last, the interval is longer than 4 months after the first time discharge from our institution.

The DS was performed by our chief surgeon, Dr. Yunzhao Zhao, MD and PhD. During the DS, a lateral-lateral end anastomosis was performed in each fistula using a linear stapler (Pride Medical Inc., Jingjiang, Taizhou, Jiangsu, China). In addition, serosa and muscularis injuries were sutured using a 4-0 absorbable band (Vicryl Plus, Ethicon, Inc., TX, USA). Before anastomosis, the digestive tract was gradually dissociated. In all cases, intra-intestinal splinting was carried out before abdominal closure. In addition to the closure of the fistula(s), hernia repair was performed for each patient during the DS. Besides, component separation technology was applied and onlay mesh repair was carried out. In this process, a Cook Biodesign advanced tissue repair device (Cook Medical Inc., Bloomington, IN, USA) was employed. Negative pressure drainage took place under all incisions.

### Postoperative Complications

The postoperative complications were defined and classified in the way as proposed by Clavien-Dindo (11), et al. In brief, the complications were classified into five levels. Any deviation from the normal postoperative course without needing the pharmacological treatment or surgical, endoscopic, and radiological interventions was classified as level I. When the pharmacological treatment with the drugs other than those permitted for level I complications was required, it was classified as level II. When surgical, endoscopic, or radiological intervention was required, it was classified as level III. Life-threatening complication (such as, CNS complications) or the need for IC/ICU management was classified as level IV. The death of a patient was classified as level V. The complications severer than level III were defined as major ones.

### Data Analysis

The baseline data were collected upon admission for the DS, such as general patient information (gender, BMI, and age), fistula output (the output was calculated according to the fistula with the maximum outflow when the patients had more than one small intestinal fistula), the area of planned ventral hernia, and such laboratory test results as WBC count, hemoglobin (Hb), and albumin (Alb). Fistula characteristics were collected according to the latest gastroenterographic data. The gastroenterography of patients was reviewed and computer-aided measurement tools were applied to determine the length from duodeno-jejunal flexure to the location of (the first) fistula, and that of the small intestine. The laboratory test was performed on a daily basis within 7 days after DS, before the results were collected and referenced for analysis. Postoperative ileus was defined as a longer defecation time than 7 days after DS.

All statistical analyses were conducted using the SPSS 26.0 software (IBM, Analytics, Armonk, NY, USA). Continuous data are described using median (interquartile range, IQR). Besides, Mann–Whitney *U*-test was performed to compare continuous variables across various groups. Fisher's exact test was carried out to compare categorical variables. Followed by a log-rank test and the multivariate Cox regression analysis, Kaplan–Meier estimates were referenced to compare the effects produced by different methods. A 1:1 propensity score-matching (PSM) was used to reduce the impact of treatment-related bias on the practice of estimating the treatment effects with observational data. The patients in the PSM groups were matched on the basis of calculated propensity scores by a regression model with demographic data, fistula characteristics, perioperative characteristics, comorbidity as covariates. The match tolerance was set to 0.1. A value of *p* < 0.05 was treated as statistically significant.

## Results

### Population and Baseline Characteristics

**Figure 1:**
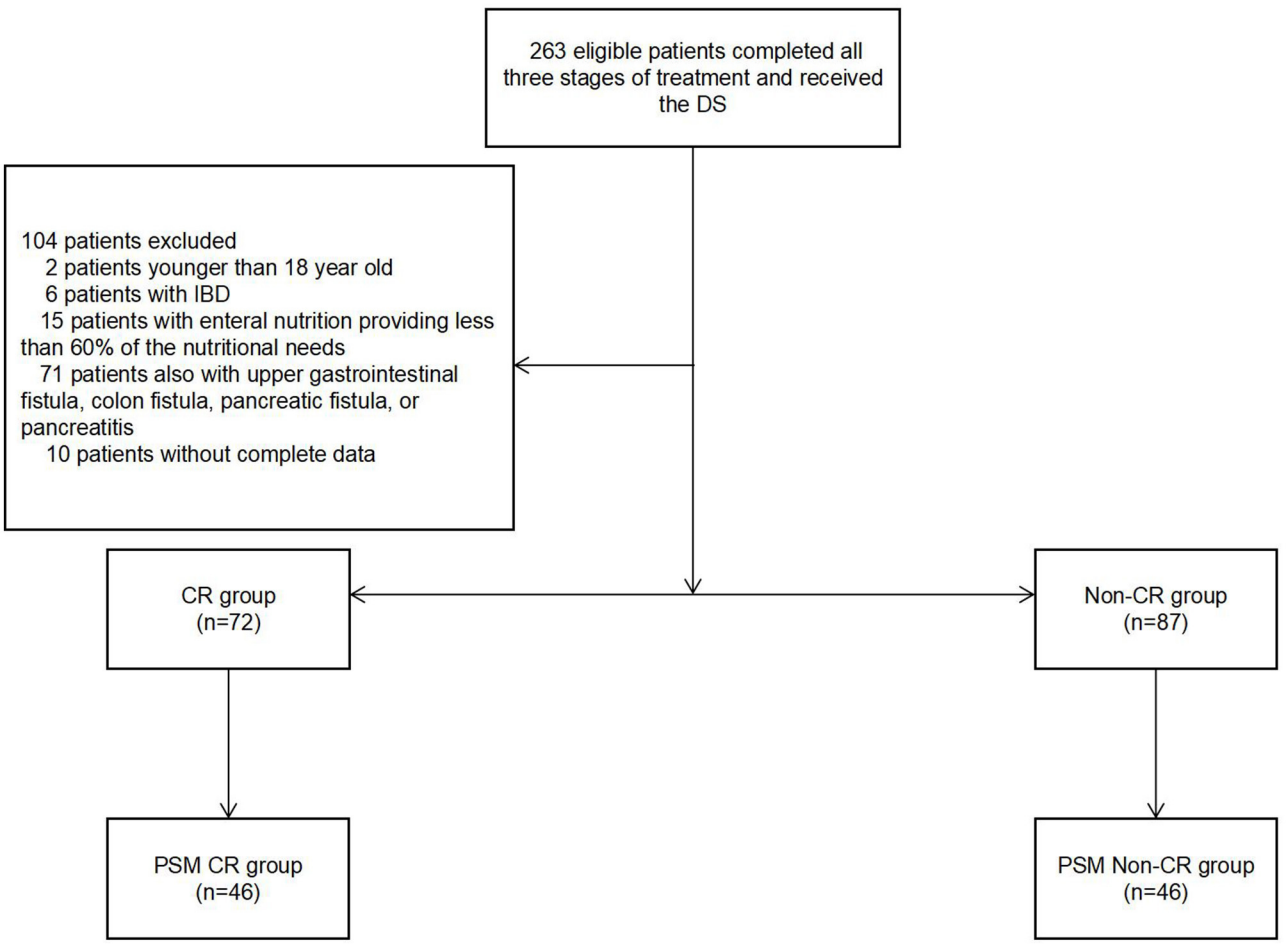
The patients and grouping.

From January 2012 to December 2019, a total of 263 eligible patients completed all three stages of the treatment while receiving the DS. A total of 159 patients were recruited in our study (Figure 1). Among them, 87 patients met the surgical criteria without CR. There were 60 (68.9%) out of the 87 patients requiring extra parenteral nutrition (PN) in their local hospital, so that they were assigned into the non-CR group. The reasons why CR was excluded from analysis is shown in Table 1.

**Table 1:** Reasons why not CR.

	**NO**.
Reasons why not CR	
Process is too complex and the manpower are not enough in local hospital	13
Psychologically unacceptable	4
Local doctors believe that without CR, the DS standard may still be reached after EN+PN or exclusive EN	26
Process is too complex and the manpower are not enough in local hospital. AND Local doctors believe that without CR, the DS standard may still be reached after EN+PN or exclusive EN.	36
Local doctors believe that without CR, the DS standard may still be reached after EN+PN or exclusive EN. AND Psychologically unacceptable.	8

Following PSM, the postoperative complications were manifested in 67 out of the 92 patients. To be specific, there were 26 patients in the PSM CR group, and 41 patients in the PSM non-CR group. CR was associated with postoperative complications (multivariate *OR* = 0.161; 95% *CI*: 0.040–0.591; *p* = 0.002, Table 6). There were eight patients in each group manifesting major complications. The occurrence of major complications was irrelevant to CR (multivariate *OR* = 1.000; 95% *CI*: 0.340–2.939; *p* = 1.000).

Table 2 shows the characteristics of these 159 patients. There were 83 women, of whom 39 were in the CR group and 44 were in the non-CR group, and *p* = 0.652. The average age was 45.0 years (IQR = 34.0–54.75 years) in the CR group, and it was 48.0 (*IQR* = 36.0–59.0 years) in the non-CR group (*p* = 0.117). The BMI was found to be comparable between the two groups (19.0 [IQR = 18.3–20.1] in the CR group, and 19.0 [IQR = 18.3–20.0] in the non-CR group *p* = 0.710). The proportion of patients with an output of less than 500 ml, between 500 and 1,000 ml, or more than 1,000 ml varied between these two groups (3, 39, and 30 in CR group and 22, 52, and 13 in Non-CR group, respectively, *p* < 0.001, Table 2).

**Table 2:** Patients characteristics.

**Clinical variables**	**Before PSM**			**After PSM**		
	**CR group (*n =* 72)**	**Non-CR group (*n =* 87)**	***p***	**PSM CR group (*n =* 46)**	**PSM Non-CR group (*n =* 46)**	***P***
Demographic data						
Female, No. (%)	39 (54.2)	44 (50.6)	0.652	24 (52.2)	23 (50.0)	0.835
Age, years; (median, IQR)	45.0 (34.0–54.75)	48.0 (36.0–59.0)	0.117	44.0 (35.0–53.5)	48 (38.0–59.2)	0.156
BMI, (median, IQR)	19.0 (18.3–20.1)	19.0 (18.25–20.0)	0.710	19.0 (18.0–19.9)	19.0 (18.0–19.8)	0.407
Fistula characteristics						
Interval from fistula occurred to admission, day, (median, IQR)	15 (12–19)	15 (12–19)	0.880	15 (12–19)	15 (12–19)	0.411
The length of stay in the local medical institutions after discharge from our institution for the 1st time, month, (median, IQR)	5 (4–6)	5 (5–6)	0.124	5 (4–6)	5 (5–6)	0.172
High intestinal fistula, No. (%)			0.018			1.000
Yes	5 (6.94)	0 (0)		0 (0)	0 (0)	
No	67 (9.31)	87 (100)		46 (100)	46 (100)	
Length of small intestine, No. (%)			0.111			0.797
<300 cm	17 (23.61)	12 (13.79)		9 (19.6)	10 (21.7)	
≥300 cm	55 (76.39)	75 (86.21)		37 (80.4)	36 (78.3)	
Duration of CR, day (median, IQR)	152 (118–182)	12 (10–16)	<0.001	154 (112–178)	14 (10–17)	<0.001
Output, No. (%)			<0.001			0.451
<500 ml/day	3 (4.17)	22 (25.29)		3 (6.5)	6 (13.0)	
≥500 ml/day, and <1000 ml/day	39 (54.17)	52 (59.77)		33 (71.7)	28 (60.9)	
≥1000 ml/day	30 (41.67)	13 (14.94)		10 (21.8)	12 (26.1)	
The area of planed ventral hernia, No. (%)			0.363			0.863
<50 cm^2^	7 (9.72)	13 (14.94)		6 (13.0)	7 (15.2)	
≥50 and <100 cm^2^	48 (66.67)	60 (68.97)		29 (63.1)	30 (65.2)	
≥100 cm^2^	17 (23.61)	14 (16.09)		11 (23.9)	9 (19.6)	
Etiology, No. (%)			0.841			0.957
Trauma	49 (68.1)	56 (64.4)		29 (63.0)	28 (60.9)	
Unclear perforation	2 (2.8)	2 (2.3)		1 (2.2)	1 (2.2)	
Obstruction	14 (19.4)	22 (25.3)		12 (26.1)	14 (30.4)	
Mesenteric thrombosis	7 (9.7)	7 (8.0)		4 (8.7)	3 (6.5)	
Perioperative characteristics						
Hemoglobin before DS, No. (%)			0.211			0.165
<120 g/L	19 (26.4)	31 (35.6)		10 (21.7)	16 (34.8)	
≥120 g/L	53 (73.6)	56 (64.4)		30 (65.3)	36 (78.2)	
Albumin before DS, No. (%)			0.172			1.000
<35 g/L	20 (27.8)	33 (37.9)		13 (28.3)	13 (28.3)	
≥35 g/L	52 (72.2)	54 (62.0)		33 (71.7)	33 (71.7)	
Duration of DS, No. (%)			0.645			0.404
≥2 h and <4 h	33 (45.8)	38 (43.7)		20 (43.5)	24 (52.2)	
≥4 h and <6 h	39 (54.2)	49 (56.3)		26 (56.5)	22 (47.8)	
Bleeding loss during DS, No. (%)			0.932			0.445
<1000 ml	12 (16.7)	13 (14.9)		10 (21.7)	9 (19.5)	
≥1000 ml and <1500 ml	48 (66.6)	58 (66.7)		27 (58.7)	32 (69.6)	
≥1500 ml	12 (16.7)	16 (18.4)		9 (19.6)	5 (10.9)	
The amount of red blood cell suspension input during DS and 48 h after DS^*^, No. (%)			0.531			0.674
<10U	30 (41.7)	34 (39.1)		19 (41.3)	21 (45.6)	
≥10U	42 (58.3)	53 (60.9)		27 (58.7)	25 (54.4)	
The amount of albumin input during DS and 48 h after DS^**^, No. (%)			0.923			0.834
<100 g	32 (44.4)	38 (43.7)		20 (43.5)	21 (45.6)	
≥100 g	40 (55.6)	49 (56.3)		26 (56.5)	25 (54.4)	
Comorbidity, No. (%)						
Hypertension	2 (2.8)	2 (2.3)	0.848	1 (2.2)	1 (2.2)	1.000
Diabetes mellitus	2 (2.8)	3 (3.5)	0.813	1 (2.2)	1 (2.2)	1.000
COPD	2 (2.8)	1 (1.2)	0.675	1 (2.2)	1 (2.2)	1.000

* *In order to maintain the Hemoglobin >100 g/L within 48 h after definitive surgery*.

** *In order to maintain the Albumin >30 g/L within 48 h after definitive surgery*.

Following PSM, 92 patients were included. To be specific, there were 46 patients each in the PSM CR group and the PSM non-CR group. The characteristics of the 92 patients between the two groups were found comparable. There were 46 women, of whom 24 were in the PSM CR group and 23 were in the PSM non-CR group, *p* = 0.835. The average age was 44.0 years (IQR = 35.0–53.5 years) in the PSM CR group, and it was 48.0 years (IQR = 38.5–59.2 years) in the PSM non-CR group (*p* = 0.156). The BMI was found comparable between these two groups (19.0 [IQR = 18.0–19.9] in the PSM CR group, and 19.0 [IQR = 18.0–19.8] in the PSM non-CR group, *p* = 0.407).

### Patients With Complications

Prior to PSM, the postoperative complications were manifested in 126 cases (79.3%). More specifically, there were forty-nine cases in the CR group, and 77 cases in the non-CR group. Among these 126 cases, there were 73 patients suffering only one complication, while the remaining 53 patients exhibited multiple complications. The complications are detailed in Tables 3, 4. CR was associated with the occurrence of postoperative complications (multivariate *OR* = 0.289; 95% *CI*: 0.123–0.733; *p* = 0.006, Table 5). There were 35 patients (22.0%) showing major complications. Of them, there were 15 patients (42.9%) in the CR group and 20 patients (57.1%) in the non-CR group. However, there was no correlation between the occurrence of major complications and CR (multivariate *OR* = 0.732; 95% *CI*: 0.349–1.538; *p* = 0.410).

**Table 3:** Characteristics of complications.

	**Before PSM**		**After PSM**	
	**CR group**	**Non-CR group**	**CR group**	**Non-CR group**
Patients with complications, *n*, (%)				
No	23 (31.9)	10 (11.5)	20 (43.5)	5 (10.9)
Yes	49 (68.1)	77 (88.5)	26 (56.6)	41 (89.1)
Number of patients with highest grade of complications, *n*, (%)				
I	8 (11.1)	5 (5.7)	10 (21.7)	5 (10.9)
II	26 (36.1)	52 (59.8)	8 (17.4)	28 (60.9)
≥III	15 (20.8)	20 (23.0)	8 (17.4)	8 (17.4)
Number of complications presented in one patient, *n*, (%)				
0	23 (31.9)	10 (11.5)	20 (43.5)	5 (10.9)
1	26 (36.1)	22 (25.29)	15 (32.6)	18 (39.1)
2	20 (27.8)	43 (49.43)	11 (23.9)	21 (45.7)
3	2 (2.8)	12 (13.79)	0	2 (4.3)
4	1 (1.39)	0	0	0

**Table 4:** Incidence of the complications.

	**CR group (*n =* 72)**	**Non-CR group (*n =* 87)**	***P***	**PSM CR group (*n =* 46)**	**PSM Non-CR group (*n =* 46)**	***P***
The overall complications, *n*, (%)	49 (68.1)	77 (88.5)	0.002	26 (56.6)	41 (89.1)	<0.001
Recurrent fistula, *n*, (%)	15 (20.8)	32 (36.8)	0.028	3 (6.5)	10 (21.7)	0.036
Postoperative ileus, *n*, (%)	27 (37.5)	67 (77.0)	<0.001	13 (28.3)	31 (67.4)	<0.001
Postoperative diarrhea, *n*, (%)	0 (0)	3 (3.5)	0.252	0	0	1.000
Gastrointestinal bleeding, *n*, (%)	0 (0)	1 (1.2)	1.000	0	0	1.000
Incisional infections, *n*, (%)	22 (30.6)	26 (29.9)	0.927	14 (30.4)	18 (39.1)	0.381
Incisional hernia, *n*, (%)	12 (16.7)	18 (20.7)	0.519	7 (15.2)	7 (15.2)	1.000

**Table 5:** Logistic regression analysis of the risk factors for postoperative complications before PSM.

**Clinical variables**	**Univariate regression**		***p***	**Multivariate regression**		***p***
	**OR**	**95%CI**		**OR**	**95%CI**	
Female	1.132	1.305–2,0.247	0.723			
CR	0.277	0.121–0.631	0.002	0.289	0.123–0.733	0.006
Age	1.009	0.983–1.034	0.509			
BMI	1.006	0.826–1.225	0.955			
Interval from fistula occurred to admission	1.016	0.945–1.092	0.669			
The length of stay in the local medical institutions after discharge from our institution for the 1st time	0.832	0.622–1.113	0.216			
Length from treitz to location of (the first) fistula						
<100 cm	Ref					
≥100 cm	0.658	0.066–5.568	0.658			
Length of small intestine						
<300 cm	Ref					
≥300 cm	0.922	0.376–2.263	0.860			
Output						
<500ml	Ref					
≥500 and <1000 ml	1.176	0.452–3.059	0.739			
≥1000 ml	1.216	0.416–3.553	0.721			
The area of planed ventral hernia						
<50 cm^2^	Ref					
≥50 and <100 cm^2^	1.400	0.510–3.847	0.514			
≥100 cm^2^	1.316	0.396–4.380	0.654			
Etiology						
Trauma	Ref					
Unclear perforation	1.375	1.138–13.720	0.786			
Obstruction	1.192	0.516–2.754	0.682			
Mesenteric thrombosis	2.750	0.582–12.990	0.202			
Hemoglobin before DS						
<120 g/L	Ref			Ref		
≥120 g/L	0.427	0.188–0.973	0.043	0.431	0.180–1.031	0.059
Albumin before DS						
<35 g/L	Ref					
≥35 g/L	0.564	0.278–1.143	0.110			
Duration of DS						
<4 h	Ref					
≥4 h	1.011	0.833–1.228	0.912			
Bleeding loss during DS						
<1000 ml	Ref			Ref		
≥1000 ml and <1500 ml	1.920	0.804–4.585	0.142	1.044	0.378–2.888	0.933
≥1500 ml	5.200	1.420–19.039	0.013	1.932	0.411–9.093	0.405
The amount of red blood cell suspension input during DS and 48 h after DS^*^						
<10 U	Ref			Ref		
≥10 U	3.466	1.706–7.042	0.001	3.098	1.301–7.380	0.011
The amount of albumin input during DS and 48 h after DS^**^						
<100 g	Ref					
≥100 g	1.589	0.797–3.167	0.188			
Hypertension	1.285	0.424–3.897	0.658			
Diabetes mellitus	1.227	0.124–12.114	0.861			
COPD	0.811	0.072–9.167	0.865			

* *In order to maintain the Hemoglobin >100 g/L within 48 h after definitive surgery*.

** *In order to maintain the Albumin >30 g/L within 48 h after definitive surgery*.

### Recurrent Fistula

Before PSM, a total of 47 (29.6%) patients were suffering from recurrent fistula after DS. Among them, there were 32 in the non-CR group, with the incidence of recurrent fistula reaching 36.8%, while the remaining 15 patients were in the CR group, with the incidence of recurrent fistula reaching 20.8%. The multivariate logistic regression was performed to reveal that the incidence of recurrent fistula in the CR (multivariate *OR* = 0.382; 95% *CI*: 0.174–0.839; *p* = 0.017) group was lower (Table 7). There were five patients receiving emergency laparotomy (*n* = 1) or puncture (*n* = 4, Table 8). Unfortunately, the patient receiving the laparotomy died on the 28th day after DS. In spite of this, among the remaining 46 patient with recurrent fistula, 38 patients underwent spontaneous closure (12 patients in the CR group and 26 patients in the non-CR group) for 39 days (IQR = 26–49 days) after DS. There were eight patients receiving an additional DS 3–6 months after the first DS (Table 8). Following PSM, a total of 13 (14.1%) patients developed recurrent fistula postoperatively. The multivariate logistic regression suggested that the incidence of recurrent fistula in the PSM CR group (multivariate *OR* = 0.223; 95% *CI*: 0.064–0.983; *p* = 0.034) was lower (Table 9). Both puncture and drainage were performed in two patients. In addition, there were four patients requiring another DS.

**Table 6:** Logistic regression analysis of the risk factors for postoperative complications after PSM.

**Clinical variables**	**Univariate regression**		***p***	**Multivariate regression**		***p***
	**OR**	**95%CI**		**OR**	**95%CI**	
Female	0.909	0.400–2.066	0.820			
CR	0.159	0.054–0.474	0.001	0.161	0.040–0.591	0.002
Age	1.006	0.962–1.037	0.692			
BMI	1.064	0.826–1.370	0.632			
Interval from fistula occurred to admission	1.006	0.917–1.103	0.904			
The length of stay in the local medical institutions after discharge from our institution for the 1st time	0.920	0.632–1.340	0.665			
Length from treitz to location of (the first) fistula						
<100 cm	Ref					
≥100 cm	1.795	0781–4.126	0.168			
Length of small intestine						
<300 cm	Ref					
≥300 cm	0.834	0.301–2.314	0.728			
Output						
<1000 ml	Ref					
≥1000 ml	0.795	0.304–2.075	0.639			
The area of planed ventral hernia						
<50 cm^2^	Ref					
≥50 and <100 cm^2^	0.511	0.123–2.122	0.356			
≥100 cm^2^	1.113	0.401–3.089	0.838			
Etiology						
Trauma	Ref					
Unclear perforation	8.172	0.554–23.599	0.819			
Obstruction	1.412	0.554–3.599	0.469			
Mesenteric thrombosis	2.589	0.464–14.461	0.278			
Hemoglobin before DS						
<120 g/L	Ref					
≥120 g/L	0.418	0.160–1.095	0.076			
Albumin before DS						
<35 g/L	Ref					
≥35 g/L	0.828	0.331–2.069	0.686			
Duration of DS						
<4 h	Ref					
≥4 h	1.174	0.516–2.670	0.702			
Bleeding loss during DS						
<1000 ml	Ref					
≥1000 ml and <1500 ml	1230	0.437–3.464	0.343			
≥1500 ml	2.778	0.640–12.059	0.173			
The amount of red blood cell suspension input during DS and 48 hours after DS^*^						
<10 U	Ref			Ref		
≥10 U	2.350	1.011–5.462	0.047	2.354	0.991–5.588	0.052
The amount of albumin input during DS and 48 h after DS^**^						
<100g	Ref					
≥100g	0.880	0.385–2.012	0.763			
Hypertension	0.837	0.051–13.797	0.901			
Diabetes mellitus	1.708	0.149–19.529	0.667			
COPD	0.837	0.051–13.797	0.901			

* *In order to maintain the Hemoglobin >100 g/L within 48 hours after definitive surgery*.

** *In order to maintain the Albumin >30 g/L within 48 hours after definitive surgery*.

**Table 7:** Multivariate logistic regression analysis of the risk factors for recurrent fistula before PSM.

**Clinical variables**	**Multivariate regression**		***p***
	**OR**	**95%CI**	
CR	0.382	0.174–0.839	0.017
Etiology			
Trauma	Ref		
Unclear perforation	3.599	0.334–38.759	0.291
Obstruction	0.502	0.188–1.336	0.167
Mesenteric thrombosis	1.107	1.256–15.464	0.021
Bleeding loss during DS			
<1000 ml	Ref		
≥1000 ml and <1500 ml	0.828	0.214–3.205	0.785
≥1500 ml	1.130	0.198–6.440	0.890
The amount of red blood cell suspension input during DS and 48 h after DS^*^			
<10 U	Ref		
≥10 U	3.174	1.157–8.706	0.025
The amount of albumin input during DS and 48 h after DS^**^			
<100 g	Ref		
≥100 g	1.338	0.514–3.478	0.551

* *In order to maintain the Hemoglobin >100 g/L within 48 h after definitive surgery*.

** *In order to maintain the Albumin >30 g/L within 48 h after definitive surgery*.

**Table 8:** Re-interventions.

	**CR group**	**Non-CR group**
Re-interventions, *n*, (%)		
Emergency laparotomy, *n*, (%)	0 (0)	1 (1.2)
Puncture and drainage	2 (2.8)	2 (2.3)
DS, *n*, (%)	1 (1.4)	0 (0)
DS + incisional hernia reparation, *n*, (%)	2 (2.8)	5 (5.8)
Incisional hernia reparation, *n*, (%)	10 (13.9)	13 (14.9)

**Table 9:** Multivariate logistic regression analysis of the risk factors for recurrent fistula after PSM.

**Clinical variables**	**Multivariate regression**		***p***
	**OR**	**95%CI**	
CR	0.223	0.047–0.884	0.034
Bleeding loss during DS			
<1000 ml	Ref		
≥1000 ml and <1500 ml	1.494	0.142–15.753	0.738
≥1500 ml	4.147	0.277–62.107	0.303
The amount of red blood cell suspension input during DS and 48 hours after DS^*^			
<10 U	Ref		
≥10 U	3.580	0.620–20.684	0.154

* *In order to maintain the Hemoglobin >100 g/L within 48 hours after definitive surgery*.

### Postoperative Ileus

**Figure 2:**
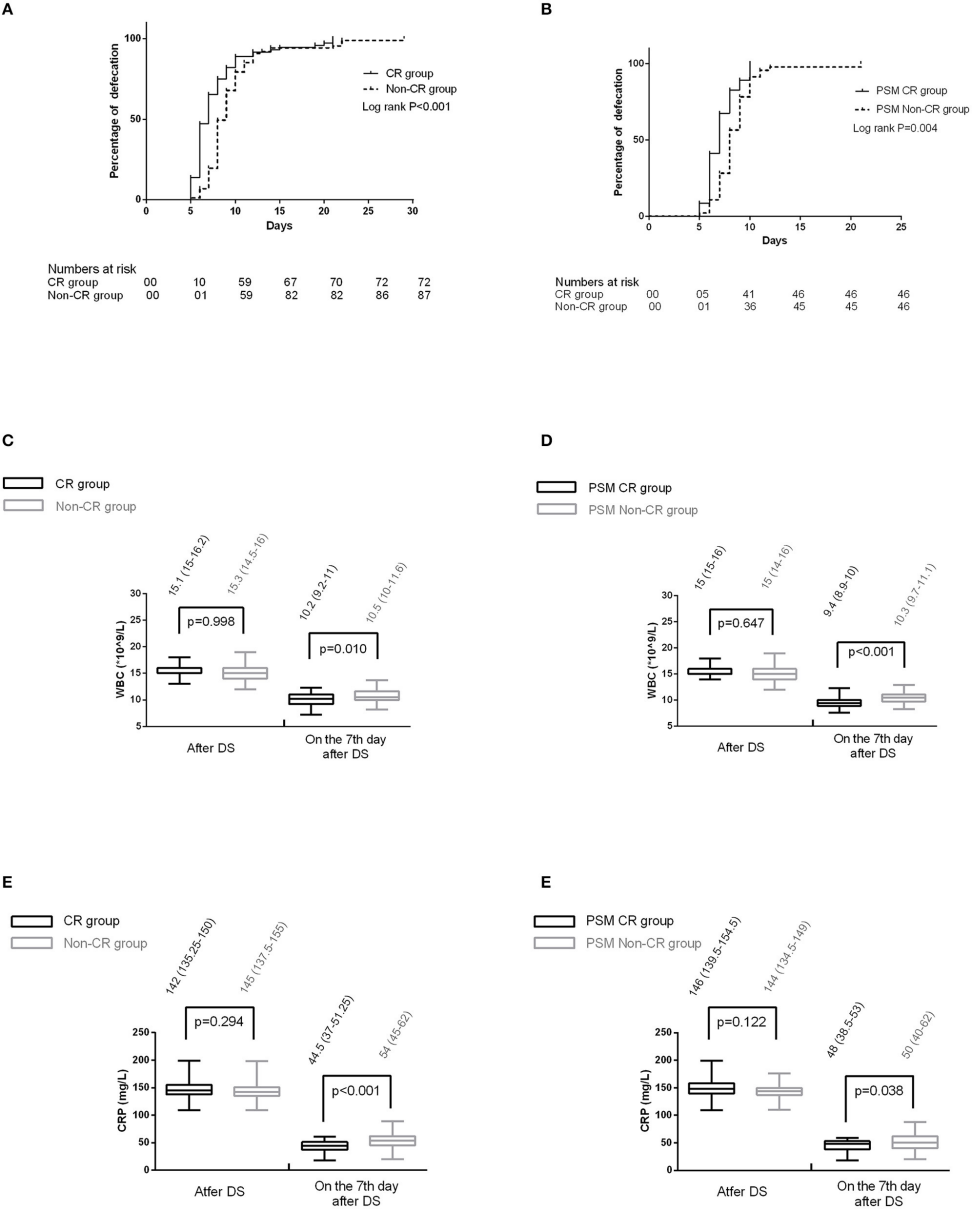
**(A)** The defecation time between the two groups before propensity score-matching (PSM); **(B)** the defecation time between the two groups after PSM; **(C)** the comparison of white blood cell (WBC) between the two groups before PSM. **(D)** the comparison of C-reactive protein (CRP) between the two groups before PSM. **(E)** The comparison of WBC between the two groups after PSM. **(F)** The comparison of CRP between the two groups after PSM.

Prior to PSM, the defecation time was 7 days (IQR = 6–8 days) in the CR group, and it was 9 days (IQR = 8–10 days, Figure 2A) in the non-CR group. The patients in the CR group accounted for 37.5% (*n* = 27), and those in the non-CR group who had postoperative ileus accounted for 77.0% (*n* = 67). CR was identified as a protective factor for postoperative ileus (multivariate *OR* = 0.209; 95% *CI*: 0.095–0.437; *p* < 0.001). There was no patient receiving surgical intervention due to postoperative ileus. Following PSM, the defecation time was 7 days (IQR = 6–8 days) in the PSM CR group and 8 days (IQR = 7–9 days, Figure 2B) in the PSM non-CR group. The occurrence of postoperative ileus was observed in 33 cases (71.7%) among the PSM non-CR group, and in 13 cases (28.3%) among the PSM CR group. According to the multivariate regression analysis, there was an association between CR and postoperative ileus (*OR* = 0.222; 95% *CI*: 0.089–0.524; *p* < 0.001).

### Incision Infections and Incisional Hernia

Before PSM, there were a total of 48 (30.2%) patients with incision-induced infection (22 in CR group and 26 in non-CR group). The impact of CR on incision infections was not detected (unadjusted *OR* = 1.032, 95% *CI*: 0.523–2.037, *p* = 0.927). Comparatively, there were 30 (18.9%) patients with incisional hernia (12 in the CR group and 18 in the non-CR group). The impact of CR on incisional hernia was not detected (unadjusted *OR* = 0.767, 95% *CI*: 0.342–1.720, *p* = 0.519) either.

Following PSM, 32 (34.8%) out of the 92 patients developed incision infections (14 in PSM CR group and 18 in non-CR group) and 16 (17.4%) out of them suffered incisional hernia (seven in the PSM CR group, and nine in the non-CR group). The impact of CR on incision infections (unadjusted *OR* = 0.681, 95% *CI*: 0.287–1.613, *p* = 0.382) and incisional hernia (unadjusted *OR* = 1.000, 95% *CI*: 0.321–3.120, *p* = 1.000) was not detected.

### Changing Trend of Postoperative Inflammatory Indexes in Non-Infected Patients

Before PSM, there were four patients that have received puncture and drainage (*n* = 3) or emergency laparotomy (*n* = 1) due to the abdominal infection caused by recurrent fistula. The remaining 154 patients exhibited no obvious signs of abdominal infection. Among these 154 patients, both the CRP and WBC decreased at a slower pace in the non-CR group (Figures 2C,D) on day 7 after DS.

After PSM, there were two patients receiving puncture and drainage. Of the remaining 90 patients, the CRP and WBC decreased at a slower pace as well in the non-CR group (Figures 2E,F) on day 7 after DS.

## Discussion

A large amount of intestinal juice leaked from the fistula hindered the flow into the distal small intestine in EAF, thus leading to a series of pathophysiologic changes in the bypassed intestine, such as villous atrophy, smooth muscle weakness, and endocrine disturbances (2, 3). Besides, it increased the risk for postoperative morbidity (9). CR is capable to reverse the pathological process and maintain the intestinal function, which is suspected to play a role in reducing complications and improving postoperative outcomes (9, 10).

The incidence of postoperative complications for complicated ECF reached almost 50% (3). In addition, the incidence of recurrent fistula and postoperative ileus exceeded 30% (3). In a small-scale study, it was shown that the incidence of complications after definitive surgery for EAF reached 100% (8/8) (12), involving three cases with recurrent fistula and six cases with hernia. This study is the first to reveal that CR is contributory in reducing the overall incidence of postoperative complications by reducing the incidence of recurrent fistula and postoperative ileus, despite CR playing no role in incision-induced complications. It is discovered in this study that the postoperative CRP and WBC in the CR group decreased at a faster pace than in the non-CR group. Therefore, it is suspected that CR is more capable to reduce the inflammatory response than non-CR.

Severe abdominal adhesion formed followed previous abdominal infection and formation of frozen abdomen in the early stage of the EAF, which increased the difficulty of DS, as well as manipulation and bleeding loss during the DS. Those effects have a significant impact on the postoperative GI function and increase the inflammatory response (13, 14). Given high inflammatory response, there would be a large number of inflammatory cells invading the tissue space, which is damaging to the smooth muscle (15). The necrosis factor-α released by mast cells can inhibit the motility of the GI tract and the healing of wound (16). Additionally, the imbalance of polarized macrophages can affect the tissue repair (17) as well. Overall, these effects may translate into an increase in the incidence of complications, such as recurrent fistula and postoperative ileus. It was demonstrated that those patients receiving complex major surgery with severe bleeding and massive manipulation tended to have both long defecation time and high incidence of anastomotic leakage (5, 18, 19), which appears to be consistent with the above theory. However, in some literature, the incidence of intestinal obstruction and recurrent fistula can be reduced by the reasonable reconstruction of GI physiological function. For those patients with EAF, CR is essential for the restoration of GI function. It reverses the pathological changes of long-term disuse while maintaining the small intestinal mucosa and function (9, 10), which might alleviate the immune and inflammatory response after DS for EAF. A surgery can have adverse effect on the patients (20).

It is suspected that there are other reasons for the advantage of CR in prognosis. For instance, CR makes a positive difference to the nutritional status (4, 5), which might improve the post-DS outcomes. However, in our study, all patient indicators must fall within the normal range before a DS could be performed for the non-CR group. As a result, the impact of CR on the nutritional improvement might not be a significant influencing factors in the occurrence of complications. In addition, CR is contributory to strengthen the smooth muscle tissue, for which the small intestine at the distal end of the fistula is more robust compared with the non-CR group. Theoretically, the most immediate impact is that the operation is easier to perform. In addition, it makes the postoperative intestinal movement near and distal to the anastomosis more coordinated, thus reducing the defecation time. Therefore, complications might be reduced.

The major complications were irrelevant to CR, as revealed in this study. This is because the surgical intervention was not required for the vast majority of patients with recurrent fistula (three patients with emergency intervention, one patient with DS, and seven patients with DS + incisional hernia reparation). In contrast, the surgical intervention was required for postoperative incisional hernia (seven patients with DS + incisional hernia reparation and 23 patients with incisional hernia reparation). In our study, CR made no difference to the incidence of hernia, because it had no impact on abdominal extension and fascia loss, etc.

There are some limitations of our study. First, this is a retrospective study, for which selection bias was likely to arise. However, the 1:1 PSM was used and a further analysis was conducted. Second, the non-CR group was not exactly a CR-naive control group, but a group with less than 1 month of CR. Therefore, it is possible that the difference in results was made more significant. Third, there is a failure to fully reveal the mechanisms behind the advantage of CR in prognosis. Therefore, a prospective collection of complementary data, from the same patients at several points of hospitalization, would be conducive to better understanding the underlying mechanisms in the future. Since the patients were as their own controls, the heterogeneity of the situations would be made less important. Besides, the follow-up time was insufficient, and the incidence of fistula was potentially biased. That is to say, some fistula may not become apparent until a certain point after discharge from the hospital. Those in the CR group were hospitalized for a significantly shorter period of time than the non-CR group (mean of 16 vs. 30 days), as a result of which the rate of recurrent fistula among them might be falsely lower.

## Conclusion

The CR performed before DS for EAF might contribute in reducing the incidence of postoperative complications.

## Data Availability Statement

The original contributions presented in the study are included in the article/supplementary files, further inquiries can be directed to the corresponding author/s.

## Ethics Statement

The studies involving human participants were reviewed and approved by Ethics Committee of Jinling Hospital. The patients/participants provided their written informed consent to participate in this study.

## Author Contributions

YZ and WT provided research objects. XX and RZ collected and analyzed the data. ZY, XX, and WT wrote the main manuscript text. XX prepared figures and revised the manuscript. ZY revised the manuscript and designed the research. All authors contributed to the article and approved the submitted version.

## Conflict of Interest

The authors declare that the research was conducted in the absence of any commercial or financial relationships that could be construed as a potential conflict of interest.

## Publisher's Note

All claims expressed in this article are solely those of the authors and do not necessarily represent those of their affiliated organizations, or those of the publisher, the editors and the reviewers. Any product that may be evaluated in this article, or claim that may be made by its manufacturer, is not guaranteed or endorsed by the publisher.
